# Role of ECLS in Managing Post-Myocardial Infarction Ventricular Septal Rupture

**DOI:** 10.3390/jcdd10110446

**Published:** 2023-10-30

**Authors:** Rodrigo Sandoval Boburg, Stoyan Kondov, Mladen Karamitev, Christian Schlensak, Rafal Berger, Helene Haeberle, Walter Jost, Albi Fagu, Friedhelm Beyersdorf, Maximilian Kreibich, Martin Czerny, Matthias Siepe

**Affiliations:** 1 Department of Thoracic and Cardiovascular Surgery, University Hospital Tübingen, 72076 Tübingen, Germany; 2 Department of Cardiovascular Surgery, University Heart Center Freiburg-Bad Krozingen, 79106 Freiburg, Germany; 3 Medical Faculty, Albert-Ludwigs-University Freiburg, 79106 Freiburg, Germany; 4 Department of Anesthesiology and Critical Care Medicine, University Hospital Tübingen, 72076 Tübingen, Germany; 5 Department of Heart Surgery, Cardiovascular Center, Inselspital, 3010 Bern, Switzerland

**Keywords:** ECLS, myocardial infarction, ventricular septal rupture

## Abstract

Objectives: The aim of this study was to analyze outcomes in patients undergoing surgery for ventricular septal rupture (VSR) after myocardial infarction (MI) and the preoperative use of extracorporeal life support (ECLS) as a bridge to surgery. Methods: We included patients undergoing surgery for VSR from January 2009 until June 2021 from two centers in Germany. Patients were separated into two groups, those with and without ECLS, before surgery. Pre- and intraoperative data, outcome, and survival during follow-up were evaluated. Results: A total of 47 consecutive patients were included. Twenty-five patients were in the ECLS group, and 22 were in the group without ECLS. All the ECLS-group patients were in cardiogenic shock preoperatively. Most patients in the ECLS group were transferred from another hospital [n = 21 (84%) vs. no-ECLS (n = 12 (57.1%), *p* = 0.05]. We observed a higher number of postoperative bleeding complications favoring the group without ECLS [n = 6 (28.6%) vs. n = 16 (64%), *p* < 0.05]. There was no significant difference in the persistence of residual ventricular septal defect (VSD) between groups [ECLS n = 4 (16.7%) and no-ECLS n = 3 (13.6%)], *p* = 1.0. Total in-hospital mortality was 38.3%. There was no significant difference in in-hospital mortality [n = 6 (27.3%) vs. n = 12 (48%), *p* = 0.11] and survival at last follow-up between the groups (*p* = 0.50). Conclusion: We detected no statistical difference in the in-hospital and long-term mortality in patients who received ECLS as supportive therapy after MI-induced VSR compared to those without ECLS. ECLS could be an effective procedure applied as a bridge to surgery in patients with VSR and cardiogenic shock.

## 1. Introduction

Ventricular septal rupture (VSR) following acute myocardial infarction (MI) is a severe complication that affects 0.25–0.31% of the patients who suffer a MI; its mortality rate is significant, lying between 44 and 56% [1]. Acute ventricular septal rupture can lead to acute RV dysfunction, pulmonary edema, and low cardiac-output syndrome, which in turn can cause a severe, rapidly developing cardiogenic shock [1]. Several studies have shown that a longer interval between VSR and surgery raises the postoperative survival rate [2,3,4]. If cardiogenic shock ensues after VSR, some patients might deteriorate rapidly despite maximal pharmacological therapy, making it difficult to delay surgery as suggested in the literature; mechanical cardiocirculatory support (MCS) could be a useful alternative to bridge these patients to surgery [4,5,6]. There are two established mechanical cardiocirculatory support settings: the counter-pulsation intra-aortic balloon pump (IABP) and extra corporeal life support (ECLS) [5,6]. Various groups have researched the benefits of IABP in patients with cardiogenic shock (CS) and VSR [2,7]. ECLS support in patients suffering VSR was recently applied for bridging to surgery and delivered promising results in high-risk patients [4,8,9]. Although MCS would seem like an effective way to bridge these patients, it is associated with complications, with bleeding being the most common [9].

The aim of this study was to analyze outcomes in patients undergoing surgery for VSR and the preoperative use of ECLS as a bridge to surgery.

## 2. Patients and Methods

We included 47 consecutive patients who underwent open VSR repair at two centers (Freiburg and Tübingen, Germany) between 01/2009 and 06/2021. The patients were divided into two groups: those treated with ECLS as a bridge to surgery and those who did not require preoperative MCS with ECLS.

The ethics committees of the universities of Tuebingen and Freiburg approved this study with project number 385/2020BO. Due to the retrospective nature of this study, written consent was waived.

### 2.1. Preoperative Parameters

Demographic data, cardiovascular risk factors, chronic kidney disease (CKD), and chronic obstructive pulmonary disease (COPD) were recorded. Relevant preoperative data such as infarction location, percutaneous coronary intervention (PCI), intubation, the appearance of cardiogenic shock (CS), and the need for MCS were also recorded. Cardiogenic shock was defined as a systolic blood pressure < 90 mmHg refractory to fluid resuscitation with clinical and biochemical evidence of end-organ dysfunction, including altered mental status, urine output < 30/mL/h, and/or serum lactate > 2.0 mmol/L, according to recommendations from the major societies [5,6].

### 2.2. Primary and Secondary Endpoints

Our primary endpoint was in-hospital mortality. Secondary endpoints were residual VSD and complications associated with surgery.

### 2.3. Surgical Technique

#### 2.3.1. Cardiopulmonary Bypass

All operations were performed via median sternotomy using cardiopulmonary bypass (CPB). CPB was established in a standard fashion by cannulating the ascending aorta and either the right atrium or superior and inferior vena cavae, depending on the desired intracardiac access (either trans-atrial or ventricular). In both centers, cardioplegic arrest was achieved by using the Buckberg cardioplegic solution. Cardioplegic solution was applied every 20 min as long as the aorta was cross-clamped. 

#### 2.3.2. Trans-Atrial Approach

After cardioplegic arrest was achieved, an incision was made in the right atrium, and the VSD was inspected after mobilizing the tricuspid valve. After identifying the borders, closure was carried out using either bovine pericardium or a dacron patch with a polypropylene suture.

#### 2.3.3. Trans-Ventricular Approach (n = 40)

After achieving cardioplegic arrest, and in patients with an anterior VSD, an incision was made parallel to the left anterior descending artery close to the apex. In the case of an anterior VSD, we prefer to only open the left ventricle. The VSDs borders were identified, and closure was performed via a running or interrupted polypropylene suture and either bovine pericardium or a dacron patch. In some cases, especially those with posterior infarction (VSD), both ventricles were incised, and closure was carried out from both sides using the same material. After achieving closure, the ventricle was closed using felt-strengthened polypropylene sutures.

#### 2.3.4. Mechanical Circulatory Support Indication

At admission, patients were evaluated for signs of hemodynamic deterioration or CS if any were present, and the decision was made to implant an IABP from 2009–2012. After 2012, the IABP was replaced by ECLS in most cases for bridging surgery. In some cases, patients were transferred from other centers where ECLS therapy was unavailable. In those cases, an IABP was implanted before transfer. After admission to our centers, IABP was switched to ECLS. The standard approach to ECLS implantation in both centers was percutaneous femoro-femoral implantation. The ECLS that were used during this time were the Maquet Rotaflow (Maquet, Hechingen, Germany) and later the Getinge Cardiohelp Systems (Getinge, Sweden).

Postoperatively, patients were treated at the respective ICU. ECLS therapy was continued for at least 3 days postoperatively; after this time, a weaning evaluation was performed in which the ECLS pump was reduced to 1 L and then intermittently stopped. During this time, transesophageal echocardiography was performed and a pulmonary catheter was inserted to measure cardiac output (CO), LV- and RV-function, cardiac index (CI), and catecholamine rate. If a patient had a CI > 2.1 L/min/m [2], catecholamine requirements with noradrenaline < 0.3 µg/kg/min, pulmonary wedge pressure < 15 mmHg, and adequate oxygenation parameters, the decision was made to explant the ECLS.

### 2.4. Statistical Analysis

Statistical analyses were performed using SSPS 26.0 (IBM Corporation, Armonk, NY, USA) and GraphPad Prism V 8 (San Diego, CA, USA). The normal distribution was tested using the Kolmogorov–Smirnov test. Continuous variables are reported as mean and standard deviation if they fulfill the criteria of a normal distribution; otherwise, medians with interquartile ranges are reported. Normally distributed variables were compared using the student’s *t*-test; otherwise, the Mann–Whitney U-test was used. Categorical variables were compared using the Chi-square test, and, in the case of small groups, Fischer’s exact test was applied. The Kaplan–Meier estimates analysis and log rank calculations were performed for survival.

We report our data according to the STROBE guidelines [10].

## 3. Results

Preoperative data are shown in Table 1. Female gender was more frequent in the ECLS group than in the group without ECLS: n = 16 (64%) vs. n = 6 (27.3%) (*p* = 0.01). There was no age difference between the two groups. There was no difference regarding the incidence of arterial hypertension, diabetes, smoking history, or renal insufficiency. We noted a significant difference between groups in the incidence of dyslipidemia: n = 10 (40%) of patients with ECLS and n = 15 (68.2%) of those without ECLS (*p* = 0.05). There was no difference in the incidence of COPD between groups.

ECLS group patients suffered an anterior MI more frequently n = 14, (58.3%) compared to the no-ECLS group n = 5 (23.8%) (*p* = 0.03). In these patients, the culprit lesion was in the left anterior descending artery (LAD). On the other hand, more patients without ECLS suffered a posterior MI more frequently n = 16 (72.7%) compared to n = 12 (48%) of ECLS group patients (*p* = 0.13). Here, the culprit lesion was found mostly in the right coronary artery (RCA). We detected no significant group difference in the number of patients who suffered an ST-elevation myocardial infarction (NSTEMI) and those who underwent a PCI. Twenty-one (84%) of the ECLS group patients were out-of-hospital transfers, while only n = 12 (57.1%) of patients in the no-ECLS group were transferred from another hospital. The entire group with ECLS suffered from CS. The remaining patient characteristics revealing no significant difference are depicted in (Table 1).

## 4. Preoperative Hemodynamic Parameters

Mean arterial pressure (MAP) at the time of arrival showed no difference between groups. The mean heart rate (HR), although higher in the ECLS group, did not differ statistically between groups. The initial pH-value at admission differed significantly, as the ECLS group’s values were higher than the no-ECLS group’s (*p* = 0.05). Initial lactate levels were significantly higher in the ECLS group (*p* = < 0.01). Troponin T levels at admission also exhibited a significant difference between groups, with the no-ECLS group having higher values (*p* = 0.04).

Up to 75% of patients in the ECLS group needed noradrenaline (NA) therapy at admission, compared to 28.6% of patients in the no-ECLS group (*p* = < 0.01). There was a significant difference between groups in adrenaline use. Approximately 45.8% of patients in the ECLS group needed adrenaline at the time of admission, compared to only 4.8% of those in the group without ECLS (*p* = < 0.01). However, their need for dobutamine did not differ (*p* = 0.45) (Table 2).

### 4.1. Intraoperative Management

Some patients underwent concomitant surgeries in addition to VSD closure; these procedures are listed in Table 3. A total of n = 4 patients who underwent interventional VSD closure required emergency surgery due to displaced occluders. Two different approaches were used to close the VSD closure: trans-atrial and trans-ventricular. The use of the trans-ventricular approach was significantly higher in the ECLS group (*p* = 0.04). All patients underwent VSD closure using either the single patch or sandwich technique; the difference between the two techniques and groups was not statistically significant (*p* = 1.0). We found no significant difference in VSD size between groups (*p* = 0.3) (Table 3). 

### 4.2. Postoperative Outcome

**Figure 1:**
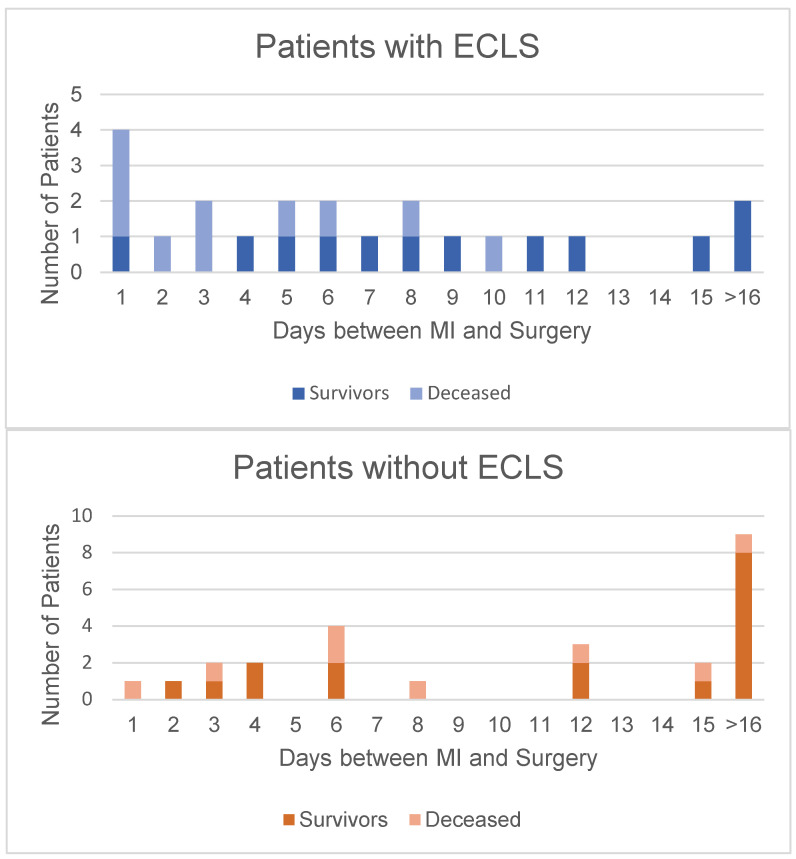
Correlation between time to surgery after diagnosis and mortality. It is evident that in both groups, a longer time interval between MI and surgery translated into higher survival rates. This is marked in the ECLS group (above) after day 7. In the group without ECLS (below), it is evident that most patients underwent surgery over 2 weeks after their initial MI.

Figure 1 shows the time from diagnosis to surgery and the corresponding mortality. The mortality rate of patients undergoing surgery within the first week after MI diagnosis, although not statistically significant, was higher in the ECLS group (*p* = 0.19). The mortality rate decreased in both groups in patients who underwent surgery after day 8 after MI diagnosis; there was no significant difference between groups (*p* = 1.0).

As Table 4 shows, the LOS in the ICU was significantly longer in patients with ECLS, with a median of 16 days compared to 8 days in the no ECLS group (*p* < 0.01). This trend was also evident in the duration of mechanical ventilation, where patients with ECLS had significantly longer ventilation support (*p* < 0.01). The number of patients who suffered from postoperative pneumonia requiring a tracheotomy did not differ significantly between groups. The rate of postoperative bleeding did differ significantly, favoring the no-ECLS group (28.6 vs. 64%) (*p* < 0.05). The number of patients whose chest was open after surgery was significantly higher in the ECLS group (*p* = 0.04). There was no difference regarding residual VSD between groups (*p* = 0.89). We found no significant difference in the number of patients who underwent re-do surgery for VSD closure (*p* = 1.0). A significantly higher number of patients in the ECLS group required postoperative dialysis (*p* = 0.05). There was no significant difference regarding postoperative wound infection between groups (*p* = 0.1). Patients had a median of 5.4 postoperative days on ECLS therapy. Overall, the in-hospital death rate was 38.3%. Although the ECLS group’s death rate was numerically higher n = 12 (48.0%) than the no-ECLS group’s n = 6 (27.3%), the difference was not significant (*p* = 0.11) (Table 4).

### 4.3. Follow-Up

**Figure 2:**
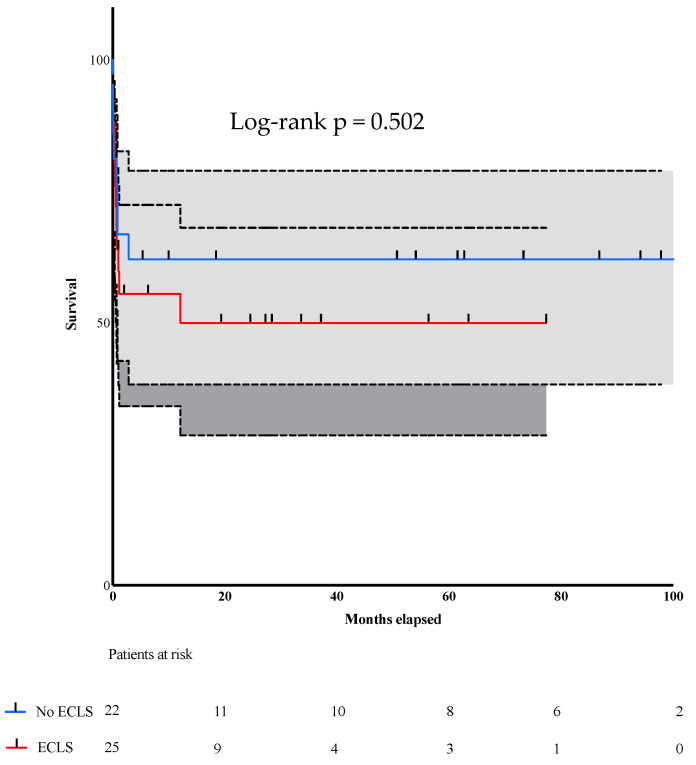
Kaplan–Meier curve depicting the survival rate.

Our long-term follow-up with these patients aimed to compare survival after surgical treatment. Figure 2 depicts the number of deceased patients during follow-up. The no-ECLS group patients had a longer follow-up because they underwent surgery earlier. Nevertheless, overall long-term survival did not differ between groups (*p* = 0.50). (Figure 2).

Figure 2: Long-term follow-up and mortality of patients after undergoing surgery for VSR after MI.

## 5. Discussion

This study analyzed the postoperative outcomes of 47 consecutive patients in two different centers in Germany who suffered from post-MI VSR. Patients were separated into two groups according to whether they received MCS with ECLS. There were 25 patients in the ECLS group and 22 in the group without ECLS. The patients who underwent ECLS therapy were in preoperative cardiogenic shock. Aside from ECLS, IABP was used in patients who were referred from other centers (in Germany, not all centers have the capability of treating patients with ECLS therapy, so patients have to be transferred to tertiary centers if this therapy is needed) in which ECLS therapy is not available; there were no other MCS strategies used in these patients. Due to the short time between VSR and either surgery or ECLS implantation in most patients, there was no ventricular unloading used.

Demographically speaking, we identified a significantly higher number of female patients in the ECLS group. The other demographic parameters revealed no significant differences. Our analysis of cardiovascular risk factors showed a significant difference in the prevalence of dyslipidemia: its prevalence in the no-ECLS group was significantly higher than in the ECLS group, but this is a coincidental finding. There were no differences in any other cardiovascular risk factors.

Infarct location differed significantly between groups. ECLS group patients suffered mainly anterior infarctions, while patients without ECLS suffered mainly posterior infarctions. Although the reports in the literature so far suggest that patients suffering a posterior infarction have worse postoperative outcomes caused by possible right ventricular dysfunction and more difficult defect closure than is the case with an anterior defect, we were unable to replicate such findings here [11,12,13,14].

Interestingly, the number of patients transferred to our centers from another hospital was significantly higher in the ECLS group than the no-ECLS group (*p* = 0.05).

When comparing preoperative hemodynamic parameters, we found that the signs of CS were most obvious in the ECLS group. There was a significant difference in troponin levels between groups at admission, which may be attributable to the longer time interval between MI and either transfer or surgery in patients without ECLS, thus having enough time for troponin levels to rise.

The preoperative parameters showed that our no-ECLS patients were in more stable condition than our patients with ECLS, an often reported characteristic [3,9,15,16,17,18,19,20]. Nevertheless, after establishing ECLS as a bridge to surgery, over half of these patients survived—an acceptable outcome knowing that the patients needing ECLS were suffering from severe CS. This is of utter importance since the postoperative outcome is strongly influenced by the preoperative state of these patients and the time between MI and surgery. Despite these differences, we detected no difference in our patient cohort’s primary outcomes—in contrast to what has been reported to date [4,13,16,20,21,22].

Our data show no differences in surgical techniques or concomitant surgeries. The decision to perform the VSD closure using a trans-atrial or trans-ventricular approach was made by the treating surgeon based on his expertise. There were no differences in closure rates or complications between both techniques. CABG did not seem to influence the primary outcome greatly, and we identified no survival benefit closely associated with CABG (*p* = 1.0), a finding that concurs with those in the current literature [23,24]. It is important to note that all four patients who underwent interventional VSD closure required emergency surgery because of occluder displacement, and only one of them died. These findings also concur with the literature, since there have been studies that describe high closure rates and low mortality [25,26]. The only patient that died had undergone an intervention and surgery one day after his MI diagnosis.

The LOS in the ICU and MV were directly influenced by the patients’ preoperative status. As expected, the ECLS group required longer MV therapy and remained longer in the ICU.

The rate of postoperative bleeding needing surgical evacuation and an open chest was higher in ECLS group patients, probably due to the heparinization needed for ECLS therapy; also, the rate of postoperative dialysis was higher in these patients—a finding widely described by others [8,9,16,22]. It is, however, important to note that the benefit from ECLS outweighs the bleeding risk, as the primary outcome and long-term follow-up in our study show. There was no significant difference in other postoperative complications.

We observed no significant difference in the residual VSD or re-do surgery rate. Only 8.0% of patients in the ECLS group died from postoperative complications after re-so surgery, whereas none of the non-ECLS patients did.

Our study’s primary outcome was in-hospital death. The major causes of in-hospital death were multiorgan failure, right ventricular failure, and low-cardiac output syndrome. Although the no-ECLS group’s death rate was lower than the ECLS group’s (27.3% vs. 48.0%), it was not statistically significant (*p* = 0.11). This parameter assumes more importance when one analyzes the ECLS group’s preoperative status: they presented with refractory CS, needed a preoperative IABP or ECLS, and, due to signs of cardiac- and multiorgan failure, urgent surgery could sometimes not be delayed long enough, as suggested in the literature [1,2,3,21,22]. Figure 1 illustrates the ECLS group patients operated on within 7 days after MI, compared to those in the no-ECLS group; the former’s mortality rate was 60% and the latter’s 25% (*p* = 0.18). The mortality rate of the patients who underwent surgery later than the 7th day after MI fell to 20% in the ECLS group and rose to 30% in the no-ECLS group (*p* = 1.0). This finding shows consistency with other groups’ results [1,3,9,16,21,22].

These results accentuate the importance of ECLS therapy as a bridge to surgery and postoperative support for these patients. The observed overall mortality was compared favorably with other published series, and we believe the preparative use of ECLS in order to stabilize and postpone patients before surgery may be a cornerstone of the good results [1,22].

The longest follow-up time was 6.66 years in the ECLS group and 8.33 years in the group without ECLS, a difference attributable to when ECLS therapy was implemented: around 2011 in both centers. Just like our study’s primary outcome, long-term mortality did not differ statistically between the groups despite the challenges that our patients and medical teams encountered during their treatment.

## 6. Study Limitations

One of the major study limitations is its retrospective nature. Another major limitation is the rareness of this condition, which makes it difficult to recruit a large cohort. Additionally, the knowledge and experience gained over the years regarding the use and implantation of ECLS may have influenced the indication for implantation during this study period. The same aspect must be considered regarding the surgical technique.

## 7. Conclusions

ECLS therapy is a safe and effective procedure that may be applied as a bridge to surgery in patients suffering from cardiogenic shock after a VSR. Despite the still high mortality rate, in-hospital death or survival during follow-up is comparable between patients with preoperative cardiogenic shock and ECLS therapy and patients without cardiogenic shock and ECLS therapy. 

## Figures and Tables

**Table 1:** Preoperative clinical parameters.

	Overall n = 47	No ECLS n = 22	ECLS n = 25	*p*-Value
Female	21 (44.7%)	6 (27.3%)	16 (64%)	0.01
Age (y)	63.5 (57.0–75.0)	67 (55.5–77)	63.5 (57.5–69.5)	0.69
Arterial hypertension	29 (61.7%)	14 (66.7%)	15 (60%)	0.77
Diabetes	7 (14.9%)	2 (9.5%)	5 (20.0%)	0.3
Smoking history	23 (48.9%)	13 (61.9%)	10 (40%)	0.18
Renal Insufficiency	10 (21.3%)	4 (19.0%)	6 (24.0%)	0.73
Dyslipidemia	24 (51.1%)	15 (71.4%)	9 (36.0%)	0.05
COPD	5 (10.6%)	2 (9.5%)	3 (12.0%)	1.00
Infarction Region:				
Anterior	19 (40.4%)	5 (23.8%)	14 (56.0%)	0.03
Posterior	28 (59.6%)	16 (72.7%)	12 (48.0%)	0.13
STEMI	33 (70.2%)	16 (84.2%)	17 (68.0%)	0.72
PCI	38 (80.1%)	17 (81.0%)	21 (84.0%)	0.71
Transport from other hospital	33 (70.2%)	12 (57.1%)	21 (84.0%)	0.05
Cardiogenic shock at admission	29 (61.7%)	4 (18.2%)	25 (100.0%)	<0.01
CPR in extern hospital	7 (14.9%)	2 (9.5%)	5 (20.0%)	0.3
IABP	21 (44.7%)	12 (54.5%)	9 (36.0%)	0.37
Days elapsed from Coronarography to Surgery	9 (5–16)	12 (6.5–35.5)	7 (4–12.6)	<0.01

**Table 2:** Preoperative hemodynamic parameters.

	Overall n = 47	No ECLS n = 22	ECLS n = 25	*p*-Value
MAP (mmHg) at admission	76.0 ± 16.1	80.6 ± 14.1	72.9 ± 16.9	0.12
HR (beats/min) at admission	99.3 ± 24.9	93.7 ± 17.5	103.2 ± 28.8	0.22
pH	7.35 ± 0.10	7.34 ± 0.12	7.40 ± 0.1	0.05
Lactate at admission (mmol/L)	2.5 (1.6–4.7)	1.6 (1.33–2.2)	3.5 (1.71–6.2)	<0.01
Noradrenaline at admission	25 (53.2%)	6 (28.6%)	19 (76.0%)	<0.01
Suprarenine at admission	12 (25.5%)	1 (4.8%)	11 (45.8%)	<0.01
Dobutamine at admission	23 (48.9%)	9 (42.9%)	14 (56.0%)	0.45
Troponin T (ng/L) at admission	306 (162–664)	572 (237–5487)	214 (151–426)	0.04

HR: heart rate; MAP: mean arterial pressure.

**Table 3:** Intraoperative parameters.

	Overall n = 47	No ECLS n = 22	ECLS n = 25	*p*-Value
CABG	13 (27.7%)	7 (31.8%)	6 (24.0%)	0.75
Concomitant surgery	22 (476.8%)	9 (40.9%)	13 (52.0%)	0.37
Surgical access ventricular atrial	39 (83.0%) 7 (14.9%)	16 (72.7%) 6 (27.3%)	23 (92.0%) 1 (4.0%)	0.04 -
Surgical technique Single patch Sandwich patch	35 (74.5%) 12 (25.5%)	17 (77.3%) 5 (22.7%)	18 (72.0%) 6 (24.0%)	1.00 1.00
VSD diameter length (mm)	19.3 ± 7.7	20.7 ± 7.7	18.1 ± 7.8	0.3

CABG: coronary artery bypass grafting; VSD: ventricular septal defect.

**Table 4:** Postoperative parameters.

	Overall n = 47	No ECLS n = 22	ECLS n = 25	*p*-Value
LOS at ICU (d)	11.5 (4.8–22.5)	8 (2.0–15.5)	16 (9.5–30)	<0.01
MV (d)	5 (1–15)	1 (1–4)	11.6 (6.3–18.3)	<0.01
Postoperative days on ECLS			5.4 (3.0–9.8)	
Pneumonia	24 (51.0%)	9 (40.9%)	15 (60.0%)	0.14
Tracheotomy	7 (14.9%)	1 (4.5%)	6 (24.0%)	0.1
Postoperative bleeding	22 (46.8%)	6 (28.6%)	16 (64.0%)	0.02
Open chest	11 (23.4%)	2 (9.1%)	9 (36.0%)	0.04
Residual VSD	13 (27.7%)	6 (27.3%)	7 (28.0%)	0.89
Revision for residual VSD	7 (14.9%)	3 (13.6%)	4 (16.0%)	1.0
Postoperative RRT	22 (46.8%)	7 (31.8%)	15 (60.0%)	0.05
Wound infection	12 (25.5%)	3 (13.6%)	9 (36.0%)	0.1
In-hospital death	18 (38.3%)	6 (27.3%)	12 (48.0%)	0.11

ECLS: extracorporeal life support; ICU: intensive care unit; LOS: length of stay; MV: mechanical ventilation; RRT: renal replacement therapy; VSD: ventricular septal defect.

## Data Availability

The data that support the findings of this study are available from the corresponding author, R.S.B., upon reasonable request.

## Abbreviations

CKD	chronic kidney disease
COPD	chronic obstructive pulmonary disease
CPB	cardiopulmonary bypass
CS	cardiogenic shock
ECLS	extracorporeal life support
HR	heart rate
IABP	intraaortic balloon pump
ICU	intensive care unit
MAP	mean arterial pressure
MCS	mechanical cardiocirculatory support
MI	myocardial infarction
NSTEMI	non- ST elevation myocardial infarction
PCI	percutaneous coronary intervention
STEMI	ST elevation myocardial infarction
VSD	ventricular septal defect
VSR	ventricular septal rupture

## References

[1] Moreyra A.E., Huang M.S., Wilson A.C., Deng Y., Cosgrove N.M., Kostis J.B. (2010). Trends in incidence and mortality rates of ventricular septal rupture during acute myocardial infarction. Am. J. Cardiol..

[2] Arnaoutakis G.J., Zhao Y., George T.J., Sciortino C.M., McCarthy P.M., Conte J.V. (2012). Surgical repair of ventricular septal defect after myocardial infarction: Outcomes from the Society of Thoracic Surgeons National Database. Ann. Thorac. Surg..

[3] Coskun K.O., Coskun S.T., Popov A.F., Hinz J., Schmitto J.D., Bockhorst K., Stich K.M., Koerfer R. (2009). Experiences with surgical treatment of ventricle septal defect as a post infarction complication. J. Cardiothorac. Surg..

[4] Morimura H., Tabata M. (2020). Delayed surgery after mechanical circulatory support for ventricular septal rupture with cardiogenic shock. Interact. Cardiovasc. Thorac. Surg..

[5] Van Diepen S., Katz J.N., Albert N.M., Henry T.D., Jacobs A.K., Kapur N.K., Kilic A., Menon V., Ohman E.M., Sweitzer N.K. (2017). Contemporary Management of Cardiogenic Shock: A Scientific Statement from the American Heart Association. Circulation.

[6] Vahdatpour C., Collins D., Goldberg S. (2019). Cardiogenic Shock. J. Am. Heart Assoc..

[7] Scholz K.H. (1999). Reperfusion therapy and mechanical circulatory support in patients in cardiogenic shock. Herz.

[8] Hobbs R., Korutla V., Suzuki Y., Acker M., Vallabhajosyula P. (2015). Mechanical circulatory support as a bridge to definitive surgical repair after post-myocardial infarct ventricular septal defect. J. Card. Surg..

[9] Rob D., Špunda R., Lindner J., Rohn V., Kunstýř J., Balík M., Rulíšek J., Kopecký P., Lipš M., Šmíd O. (2017). A rationale for early extracorporeal membrane oxygenation in patients with postinfarction ventricular septal rupture complicated by cardiogenic shock. Eur. J. Heart Fail..

[10] Von Elm E., Altman D.G., Egger M., Pocock S.J., Gøtzsche P.C., Vandenbroucke J.P. (2014). The Strengthening the Reporting of Observational Studies in Epidemiology (STROBE) Statement: Guidelines for reporting observational studies. Int. J. Surg..

[11] Batts K.P., Ackermann D.M., Edwards W.D. (1990). Postinfarction rupture of the left ventricular free wall: Clinicopathologic correlates in 100 consecutive autopsy cases. Hum. Pathol..

[12] Moore C.A., Nygaard T.W., Kaiser D.L., Cooper A.A., Gibson R.S. (1986). Postinfarction ventricular septal rupture: The importance of location of infarction and right ventricular function in determining survival. Circulation.

[13] Anderson D.R., Adams S., Bhat A., Pepper J.R. (1989). Post-infarction ventricular septal defect: The importance of site of infarction and cardiogenic shock on outcome. Eur. J. Cardiothorac. Surg..

[14] Cinq-Mars A., Voisine P., Dagenais F., Charbonneau É., Jacques F., Kalavrouziotis D., Perron J., Mohammadi S., Dubois M., Le Ven F. (2016). Risk factors of mortality after surgical correction of ventricular septal defect following myocardial infarction: Retrospective analysis and review of the literature. Int. J. Cardiol..

[15] Abrams D., Garan A.R., Abdelbary A., Bacchetta M., Bartlett R.H., Beck J., Belohlavek J., Chen Y.S., Fan E., Ferguson N.D. (2018). Position paper for the organization of ECMO programs for cardiac failure in adults. Intensive Care Med..

[16] Vondran M., Wehbe M.S., Etz C., Ghazy T., Rastan A.J., Borger M.A., Schroeter T. (2021). Mechanical circulatory support for early surgical repair of postinfarction ventricular septal defect with cardiogenic shock. Artif. Organs.

[17] McLaughlin A., McGiffin D., Winearls J., Tesar P., Cole C., Vallely M., Clarke A., Fraser J. (2016). Veno-Arterial ECMO in the Setting of Post-Infarct Ventricular Septal Defect: A Bridge to Surgical Repair. Heart Lung Circ..

[18] Ram E., Kogan A., Orlov B., Raanani E., Sternik L. (2019). Preoperative Extracorporeal Membrane Oxygenation for Postinfarction Ventricular Septal Defect. Innovations.

[19] Artemiou P., Gasparovic I., Bezak B., Hudec V., Glonek I., Hulman M. (2020). Preoperative extracorporeal membrane oxygenation for postinfarction ventricular septal defect: Case series of three patients with a literature review. J. Card. Surg..

[20] Matteucci M., Ronco D., Corazzari C., Fina D., Jiritano F., Meani P., Kowalewski M., Beghi C., Lorusso R. (2021). Surgical Repair of Postinfarction Ventricular Septal Rupture: Systematic Review and Meta-Analysis. Ann. Thorac. Surg..

[21] Pojar M., Harrer J., Omran N., Turek Z., Striteska J., Vojacek J. (2018). Surgical treatment of postinfarction ventricular septal defect: Risk factors and outcome analysis. Interact. Cardiovasc. Thorac. Surg..

[22] Ronco D., Matteucci M., Ravaux J.M., Marra S., Torchio F., Corazzari C., Massimi G., Beghi C., Maessen J., Lorusso R. (2021). Mechanical Circulatory Support as a Bridge to Definitive Treatment in Post-Infarction Ventricular Septal Rupture. JACC Cardiovasc. Interv..

[23] Horan D.P., O’Malley T.J., Weber M.P., Maynes E.J., Choi J.H., Patel S., Challapalli J., Luc J.G.Y., Entwistle J.W., Todd Massey H. (2020). Repair of ischemic ventricular septal defect with and without coronary artery bypass grafting. J. Card. Surg..

[24] Takahashi H., Arif R., Almashhoor A., Ruhparwar A., Karck M., Kallenbach K. (2015). Long-term results after surgical treatment of postinfarction ventricular septal rupture. Eur. J. Cardiothorac. Surg..

[25] Schlotter F., de Waha S., Eitel I., Desch S., Fuernau G., Thiele H. (2016). Interventional post-myocardial infarction ventricular septal defect closure: A systematic review of current evidence. EuroIntervention.

[26] Giblett J.P., Jenkins D.P., Calvert P.A. (2020). Transcatheter treatment of postinfarct ventricular septal defects. Heart.

